# Peer-assisted learning in critical care: a simulation-based approach for postgraduate medical training

**DOI:** 10.1080/10872981.2025.2497333

**Published:** 2025-05-05

**Authors:** Po-Wei Chiu, Shao-Chung Chu, Chia-Han Yang, Huan-Fang Lee, Hsuan-Man Hung, Hsiang-Chin Hsu

**Affiliations:** a Department of Emergency Medicine, National Cheng Kung University Hospital, Tainan, Taiwan; b Institute of Creative Industries Design, National Cheng Kung University, Tainan, Taiwan; c Department of Nursing, College of Medicine, National Cheng Kung University, Tainan, Taiwan; d Department of Nursing, Fooyin University, Kaohsiung, Taiwan; e Department of Emergency Medicine, School of Medicine, College of Medicine, National Cheng Kung University, Tainan, Taiwan

**Keywords:** Peer-assisted learning, simulation education, clinical competence, continuing medical education, team-based learning

## Abstract

Enhancing clinical competence in postgraduate year (PGY) trainees is crucial for effective patient care, especially in emergency medicine. This study investigated the impact of a well-designed, group-developed, peer-assessed learning approach combined with high-fidelity simulations on clinical skills and teamwork of PGY trainees. PGY trainees participated in a one-month program featuring team development, clinical training, scenario design, simulation, peer-assisted debriefing, and post-course evaluations at one week and three months. Trainees were divided into two groups, engaged in clinical practice, group discussions, and developed critical scenarios under mentor guidance to challenge the other group. Teamwork performance was assessed using the TEAM scale, Ottawa Global Rating Scale, and reflective essays. Follow-up evaluations employed the PGY Clinical Proficiency Evaluation scale. Trainees identified deficiencies in situation monitoring and maintaining composure, noting difficulties in effectively monitoring and reassessing situations. Despite having passed ACLS training, participants recognized their lack of clinical experience in managing critically ill patients, handling dynamic situations, low self-confidence, and limited leadership opportunities in resuscitation teams. However, team morale was high, and performance in communication and leadership was relatively strong due to the similar hierarchical levels of the trainees and initial team dynamics established during their training. Follow-up questionnaires indicated significant improvements in clinical confidence, reasoning abilities, familiarity with ACLS resuscitation guidelines, and team dynamics across various subspecialty training areas. The integration of peer-assisted learning with high-fidelity simulation significantly enhanced clinical competence, teamwork, and confidence in PGY trainees. This innovative approach provides a structured, supportive learning environment that effectively prepares trainees for real-world clinical challenges. Future research should explore long-term outcomes and broader applications of this method.

## Introduction

Over the past two decades, simulation education has become well-established and applied across various groups, including undergraduate and postgraduate medical students, nursing students, physicians, registered nurses, and paramedics. It is recognized that simulation bridges the gap between theory and practice, providing an opportunity for learners to apply their theoretical knowledge in a safe and realistic setting. It also fosters the development of teamwork and problem-solving skills [1]. Advancements in this field have evolved from simple simulations to high-fidelity, in-situ, and high-tech simulations that incorporate virtual, augmented, and mixed reality technologies.

High-fidelity simulation is a well-designed and established form of training that has become essential in emergency and critical care medicine education by emphasizing immersive and interactive experiences that diverge from traditional, educator-centric methods [2]. This approach fosters active participation and experiential learning, with participants engaging in reflective debriefing to improve self-assessment and skills [3]. Despite their advantages, most high-fidelity simulations are conducted with instructors setting learning objectives and designing lesson plans. Learners passively follow these plans and learn through feedback (debriefing) after practice.

Peer-assisted learning (PAL), also referred to as peer instruction, is a widely recognized and utilized evidence-based teaching strategy, especially in the field of physics [4]. It is recommended as one of the premier approaches in science education, with more than a quarter of university physics professors incorporating PAL into their teaching. The benefits of PAL include increased engagement and motivation, improved understanding through peer explanation and discussion, and the development of critical thinking and interpersonal skills. It also allows students to learn in a more relaxed and supportive environment, which can be less intimidating than traditional teacher-led settings [5].

This method capitalizes on the knowledge and skills of fellow students, typically with more experienced or knowledgeable students (peer tutors) assisting those who are less advanced. PAL can take various forms, such as tutoring sessions, group discussions, and collaborative projects. In medical education, PAL enables students to hone organizational, interpersonal, and teaching skills. Research indicates that 90% of students would recommend this teaching approach, and 85% felt more at ease with it [6]. The suggested benefits of PAL include fostering of a supportive learning environment and delivering instruction tailored to the learners’ needs as understood by the tutors [7]. Implementing such a teaching program requires the involvement of multiple staff members within the institution that yields a threefold benefit for students, staff, and the institution in medical education.

PAL in simulation education combines the collaborative nature of peer instruction with the practical, hands-on approach of simulation [8]. This educational strategy involves students teaching and learning from each other within simulated environments, which are designed to mimic real-life scenarios particularly in critical and emergent situations. In these settings, more experienced or knowledgeable students, often referred to as peer tutors, guide their less-experienced peers through complex simulations. This method not only enhances the learning experience by allowing students to apply theoretical knowledge in practical scenarios, but it has also been proved that PAL in simulation-based education is perceived positively by students, with the perceived benefits appearing to be greater for the peer tutors than for the peer learners [9].

Despite the growing recognition and integration of PAL with simulation-based medical education in nurses and medical students, its application in postgraduate training, particularly in high-stakes environments such as emergency medicine, remains unexplored [10,11]. Most existing studies on PAL focus on medical students or nursing trainees, with limited research investigating its role in enhancing clinical decision-making, leadership, and teamwork among postgraduate year (PGY) physicians [10,11]. Given the increasing complexity of emergency care, it is crucial to examine whether structured PAL strategies can complement traditional faculty-driven training models to better prepare PGY trainees for real-world clinical challenges.

Additionally, current simulation-based education predominantly relies on faculty-driven approaches, where instructors dictate learning objectives and scenario execution [12,13]. While effective, this model may limit trainee autonomy, engagement, and reflective learning [14,15]. Given that emergency medicine requires rapid decision-making, team coordination, and adaptability, there is a need to explore alternative training strategies that foster these competencies in PGY trainees.

This study sought to bridge these gaps by integrating structured PAL with high-fidelity simulation, allowing trainees to actively engage in scenario design, peer instruction, and collaborative learning within an immersive, real-world setting. By embedding peer-driven learning strategies within a structured simulation framework, we hypothesized that this approach would enhance the effectiveness of traditional faculty-led simulation, promote deeper cognitive processing, and improve clinical performance. Accordingly, this study was guided by the following research questions [1]: Does integrating PAL with high-fidelity simulation improve clinical competence, including confidence, reasoning ability, and familiarity with resuscitation procedures among PGY trainees? [2] How does PAL impact teamwork performance, leadership, and communication skills in emergency medicine settings? [3] What are the perceived benefits and challenges of implementing flipped simulation and peer-facilitated learning from the trainees’ perspectives?

We hypothesized that [1] PGY trainees participating in PAL-integrated high-fidelity simulation would demonstrate significant improvements in clinical confidence, reasoning ability, teamwork, and familiarity with resuscitation protocols [2]; peer-facilitated simulation would foster deeper engagement and reflective learning compared to traditional faculty-led models; and [3] trainees would perceive flipped simulation and peer-led teaching as valuable and effective components of their postgraduate training.

The objective of this study was thus to assess the impact of incorporating PAL into conventional simulation training for PGY physicians, evaluating its effects on clinical confidence, teamwork, decision-making, and leadership in emergency medicine training.

## Materials and methods

### Study design

This study was of a prospective mixed-methods design, combining both quantitative and qualitative approaches to evaluate the effectiveness of integrating PAL into high-fidelity simulation-based critical care training for PGY physicians. The study spanned a period of five months and included a detailed assessment of training outcomes through quantitative evaluation scales and qualitative reflective feedback.

### Participants

This prospective mixed quantitative and qualitative study was conducted in the affiliated hospital of National Cheng Kung University in Taiwan, from 1 August 2022, to 31 January 2023. During this period, PGY trainees assigned to the ED were enrolled in this study. Each month, about 6 to 7 PGY trainees would rotate into the ED course. The study targeted 29 PGY trainees who rotated through the ED. Before participating in this new PAL simulation curriculum, all trainees had attended either high-fidelity simulations conducted by other instructors or Advanced Cardiovascular Life Support (ACLS) training courses organized by the hospital.

### PAL simulation course

All PGYs received an orientation on their first day in the ED to understand the course proceedings. Instructors responsible for orientation divided them into two groups, with an average of 3 PGY trainees per group. The grouping was based on the principles of the ‘Resuscitation Triangle’ and ‘High-Performance Team Dynamics’ frameworks, which emphasize efficiency, structured roles, and teamwork in emergency settings [16]. These frameworks outline three hands-on roles (Compressor, AED/Monitor/Defibrillator, Airway/Ventilator) and three leadership roles (Team Leader, IV/IO/Medication Administrator, Timekeeper/Recorder). Ideally, a full resuscitation team consists of six members, but when fewer are available, individuals may need to assume multiple roles. By organizing trainees into teams of three, we aimed to simulate real-world challenges in role adaptability and teamwork while ensuring an immersive and structured learning experience.

**Figure 1.**
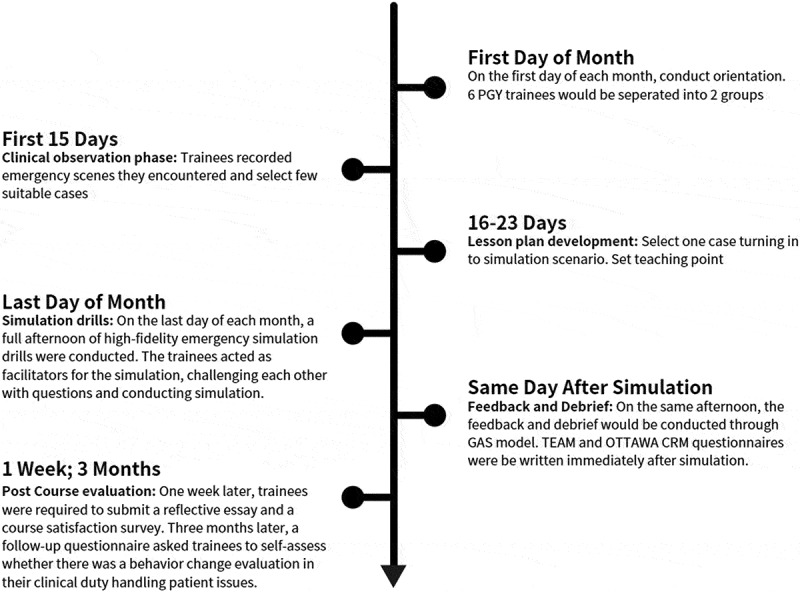
The whole PAL simulation training course.

Throughout the month, all trainees participated in four stages of training: clinical observation, lesson plan development, simulation drills, and post-course evaluation (Figure 1).

#### Clinical observation

Trainees recorded emergency scenes they encountered during their rotation in the ED. Each group discussed all of the cases they documented and selected 3 or 4 cases they deemed educationally significant for physicians at the PGY stage.

#### Lesson plan development

In this stage, in consultation with their supervising mentor, the trainees selected the most suitable case for developing a realistic critical care teaching scenario. Under mentor guidance, they collaboratively developed lesson plans that ensured comprehension of key learning points. Groups of 3 to 4 trainees discussed and compiled the content into comprehensive teaching plans. The content of each plan remained confidential and was not disclosed to the other group. To enhance interdisciplinary learning, trainees were grouped based on their future specialty interests – those inclined toward surgical specialties were assigned to the surgical group, while those interested in internal medicine were assigned to the medical group. If trainees did not have a clear preference, they were randomly assigned. Each group was then instructed to select and develop cases outside their specialty interest – meaning the surgical group designed an internal medicine case, while the medical group developed a surgical case to challenge the other group. This ‘reverse selection’ strategy provided two key benefits [1]: trainees remained engaged with cases related to their preferred specialty, and [2] as PGY trainees undergo general medical training, this approach encouraged them to study and design structured lesson plans in areas beyond their immediate specialty interest. Prior to the formal drill, each group engaged in a tabletop simulation with the mentor to ensure accuracy and smooth execution of the process on the day of the drill.

#### Simulation drills

On the last day of each month, a full afternoon of high-fidelity emergency simulation drills was conducted. The trainees acted as facilitators for the simulation, with the surgical group designing cases to challenge the medical group, and vise versa. This ‘reverse selection’ strategy ensured that trainees engaged with clinical scenarios outside their primary area of interest, promoting interdisciplinary learning and adaptability. The mentor played a supporting and guiding role to help them complete the entire simulation process. After the simulation, the group used the GAS (Gather, Analyze, Summarize) model for debriefing and watched the video playback to review mistakes that occurred during the simulation. The trainees were also required to give feedback to their peers and to prepare a 5-minute presentation to express their ideas and core clinical knowledge based on the case scenarios they developed. Further details on the execution plan, including instructional guidelines and examples, are provided in the supplementary data. (Supplementary file)

#### Post-course evaluation

After completing the simulation drills, evaluations and reflections were conducted at three intervals: on the same day, one week later, and three months later. On the day of course completion, evaluations were performed using the Team Emergency Assessment Measure (TEAM) [17], which has a scoring range from a maximum of 5 points to a minimum of 1 point, and the Ottawa Crisis Resource Management Global Rating Scale (Ottawa GRS) [18], which ranges from a maximum of 7 points to a minimum of 1 point. The TEAM scale was chosen to assess teamwork performance in emergency settings, focusing on communication, leadership, and situational awareness, while the Ottawa GRS was used to evaluate broader crisis resource management competencies, including problem-solving and decision-making. The combination of these two tools provided a comprehensive assessment of both team dynamics and individual crisis management skills.

One week later, the trainees were required to submit a reflective essay and complete a course satisfaction survey. Three months later, a follow-up questionnaire, referred to as the PGY Clinical Proficiency Evaluation (PGY-CPE) scale, was administered to assess the trainees’ self-reported behavioral changes in their clinical duties and patient management (Appendix). This scale ranges from a maximum of 5 points to a minimum of 1 point. Before commencing this course, we conducted interviews with the PGY trainees to categorize the common challenges they frequently face during on-call duties. These challenges include levels of clinical confidence, reasoning ability, familiarity with resuscitation flow, and teamwork in TRM (Team Resource Management). These categories were then used as the primary content for the pre- and post-course questionnaires we named the PGY-CPE scale.

### Simulated emergency room

A simulated emergency room was set up in a high-fidelity room at the National Cheng Kung University Guo-Ding Clinical Skills Center. The simulation was meticulously designed to replicate real-world conditions in an emergency resuscitation bay and included Laerdal SimMan Critical Care simulators, patient gowns, emergency beds, bedsheets, resuscitation trolleys, infusion tubes, ventilators, suction devices, and oxygen supply equipment.

### Data analysis

This study incorporated a mixed design method using quantitative and qualitative data.

For the quantitative data, including TEAM and CRM scale scores, descriptive statistics were presented using box-and-whisker plots. The pre- and post-intervention scores from the PGY-CPE scale were analyzed using the Mann-Whitney U test to assess the impact of peer-associated learning in the high-fidelity simulations. The data were calculated and analyzed by IBM SPSS Statistics software (version 25.0; SPSS Inc., Chicago, IL, USA)

For the qualitative data, reflective essays written from the PGY trainees were analyzed using NVivo 14 qualitative data analysis software (QSR International). To ensure data richness and authenticity, reflective writing was conducted without interference. Completed reflections were e-mailed to the corresponding author for collection. The corresponding and first author reviewed the reflections, highlighting themes in different colors, and discussing their findings. The results were then discussed with the second author to identify any conceptual errors. This triangulation method enhanced data validity, credibility and trustworthiness of the qualitative findings and strengthened their potential application in future medical education.

## Results

This course lasted for 5 months, with 10 groups of PGY participants (in which each group enrolled 2 to 3 people), and 29 trainees enrolled in the course. Among them, 14 were PGY1 trainees and 15 were PGY2 trainees. There were 11 female trainees and 18 male trainees, with an average age of 25.2 years.

### Quantitative data

In total, 29 PGY trainees participated in the course and completed the self-assessments using the Team Emergency Assessment Measure (TEAM) and Ottawa Global Rating Scale (GRS). The trainees reported notable challenges in several areas. (Table 1) On the TEAM scale, situation monitoring was rated with a mean score of 3.12 (SD = 0.78), and acting with composure and control scored a mean of 3.10 (SD = 0.72). Anticipation of potential actions was rated at 3.00 (SD = 0.70), and the ability to effectively monitor and reassess situations received a mean score of 3.34 (SD = 0.75). In contrast, higher scores were observed in team communication (mean = 3.72, SD = 0.65), leadership (mean = 3.82, SD = 0.70), and team morale (mean = 3.89, SD = 0.68).

**Table 1.** Self-assessment and follow-up evaluation scores of PGY trainees.

Assessment Tool			Mean	Standard Deviation
**1a Team Emergency Assessment Measure (TEAM) and Ottawa Global Rating Scale (GRS) Self-Assessment Scores**				
Team Emergency Assessment Measure				
*N* = 29	The team leader let the team know what was expected of them through direction and command		3.83	0.96
	Team leader maintained a global perspective		3.83	0.96
	The team communicates effectively		3.72	0.92
	The team worked together to complete tasks in a timely manner		3.48	0.87
	The team acted with composure and control		3.10	0.94
	The team morale was positive		3.89	1.01
	The team adapted to changing situations		3.34	1.14
	The team monitored and reassessed the situation		3.14	1.12
	The team anticipated potential actions		3.00	0.89
	The team prioritized tasks		3.55	0.91
	The team followed approved standards/guidelines		3.66	1.01
Ottawa Global Rating Scale				
*N* = 29	Leadership skills		5.10	1.08
	Problem solving skills		5.00	1.04
	Situational awareness skills		4.62	1.32
	Resource utilization skills		4.89	1.26
	Communication skills		5.17	1.10
	Overall performance		4.60	1.08
	Before course *N* = 29 Mean ± (SD)	Post course *N* = 18 Mean ± (SD)	Mean Difference	p value
**1b. PGY Clinical Proficiency Evaluation (PGY-CPE) Scores**				
Levels of clinical confidence	1.38 ± 0.78	4.72 ± 0.75	+ 3.33	<0.001
Reasoning ability in clinical situation	1.67 ± 0.76	4.39 ± 0.67	+ 2.72	<0.001
Familiarity with resuscitation	1.56 ± 0.78	4.11 ± 1.02	+ 3.16	<0.001
Teamwork in TRM	1.38 ± 0.50	4.44 ± 0.78	+ 3.05	<0.001

Similarly, the Ottawa GRS results reflected strengths and weaknesses. Situational awareness was rated with a mean score of 4.62, overall performance at 4.60, and problem-solving at 5.00. Communication performance, however, was rated higher, with a mean score of 5.17, indicating that trainees excelled in interpersonal interaction during simulations.

Follow-up evaluations were conducted using the PGY Clinical Proficiency Evaluation (PGY-CPE) scale three months after the course. (Table 1) Of the 29 trainees, 18 responded to this follow-up. Pre- and post-course comparisons revealed significant improvements in four key domains. Confidence levels rose markedly, with scores increasing from a pre-course mean of 1.38 (SD = 0.78) to 4.72 (SD = 0.75). Reasoning ability in clinical situations showed a substantial increase from 1.67 (SD = 0.76) to 4.39 (SD = 0.67). Familiarity with resuscitation improved from 1.56 (SD = 0.78) to 4.11 (SD = 1.02), and teamwork dynamics during team resource management (TRM) improved from 1.38 (SD = 0.50) to 4.44 (SD = 0.78). All these changes were statistically significant, with p-values less than 0.001. These findings underscore the impact of the peer-assisted learning simulation curriculum in addressing both individual and team-based skills among PGY trainees, particularly in areas critical for managing emergencies.

### Qualitative data

All 29 participants submitted follow-up reflective essays. The findings were divided into major themes: Enhancement of Clinical Performance, Value of Simulation Training, and Value of Flipped Simulation (Table 2). Overall, participants reported increased clinical confidence, a deepened understanding of emergency care teamwork, and an appreciation for the active learning process involved in scenario creation. These reflections highlight the impact of simulation-based education in bridging the gap between the theoretical knowledge and hands-on experience.

**Table 2.** Themes for PGY trainees’ reflective essays after PAL-Simulation curriculum.

Theme	Subtheme	Meaningful units
Enhancement of Clinical Performance	Application of Knowledge	- Despite having studied ACLS, practical application remains difficult (P2) - No matter how much you study, real-life emergencies still cause panic (P9)
	Resuscitation Process Handling	- Learned systematic emergency procedures through simulation courses (P18) - Trained in simulations on how to respond quickly and follow SOPs (P9)
	Increase in Confidence	- Gained clinical confidence through simulation courses (P10) - Discovered potential and improvement in emergency situations (P24)
Value of Simulation Training	Safe Learning Environment	- Simulation courses provide a safe and pressure-free learning environment (P12) - Able to learn and make mistakes in simulations without worrying about harming patients (P9)
	Practice and Reflection Importance of Teamwork and Communication	- Post-event reviews help identify shortcomings and improve (P17) - Learned how to improve one’s operations through reflection (P10) - Understood the importance of teamwork through simulation courses (P21) - Realized the importance of closed-loop communication for team operations (P3) - Lack of closed-loop communication can lead to unclear instructions (P27)
Value of Flipped Simulation	Peer-Assisted Learning	- Observed and learned from the handling methods of senior students during the course (P21) - Participated in peer-designed lesson plans and learned from them (P13) - Improved operations through peer feedback and reminders (P10) - Learned teamwork skills through collaboration with group members (P20) - Strengthened learning by posing questions and challenging each other among peers (P29)
	Challenges of Lesson Plan Design	- Designing lesson plans led to a deeper understanding of diseases (P22) - Difficulties and challenges encountered in lesson plan design (P14)
	Experience of Lesson Plan Implementation Luck and Gratitude	- Designing lesson plans led to a deeper understanding of diseases (P22) - Difficulties and challenges encountered in lesson plan design (P14) - I was very fortunate to be scheduled for the simulation course this month. (P20) - I was very lucky to be able to participate in the clinical emergency drill this time. (P11) - I was very fortunate to participate in the simulation course hosted by Dr. Chiu. (P13) - I am very grateful to Dr. Chiu for sacrificing his time to conduct this course. (P26) - Thank you, teacher, for bringing us such a fun and exciting course. (P28) - A big thank you to Dr. Chiu, the Clinical Skills Center staff, and my teammates for their help. (P4)

## Enhancement of clinical performance

Despite all trainees having completed the ACLS examination and possessing ward duty experience, it was evident that knowledge from textbooks does not compare to the profound impact of practical, hands-on experience. This was demonstrated by the trainees’ enhanced familiarity with the systematic evaluation of critically ill patients. Furthermore, their confidence in clinical practice had significantly increased.

We can better apply our everyday medical knowledge, clinical skills, decision-making abilities, and team communication in practice. Additionally, feedback from instructors and team members helps us identify blind spots and errors more effectively. We gained clinical confidence through simulation courses. (P10 male)

## Value of simulation training

For the PGY trainees, rotations in the ED typically involve managing less severe cases, with few opportunities to directly handle critical patients in the resuscitation room. This often leaves trainees feeling unfulfilled as they do not get to experience the true nature of emergency care.

After participating in a chaotic high-fidelity simulation drill, the PGY trainees truly understood the importance of teamwork. They fully grasped the critical roles of teamwork, role assignment, and closed-loop communication in emergency situations.

For PGY, it’s rare to have the opportunity to observe the initial management and assessment of critically ill patients in resuscitation bay, which I always felt was a bit unfortunate. (P12 male)

Since opportunities to participate in critical cases in the resuscitation room are rare during regular emergency department shifts, the ability to engage in safe and effective training without harming patients is especially valuable. (P15 male)

Realized the importance of closed-loop communication for team operations. (P3)

## Value of flipped simulation

Because all of the teaching cases are developed by the trainees through clinical observation and adapted from real cases with authentic data, the process of observation and case development itself becomes a learning experience. Moreover, as trainees create scenarios for each other to practice, the team creating the scenarios indirectly compares their peers’ performances with the real-life emergency team behaviors they have observed. Through this observation, trainees learn different perspectives from each other. Moreover, most trainees had a grateful attitude and considered themselves lucky to have participated in this class. Most PGY trainees who rotate through the ED do not like to join any educational classes or exert a lot of effort to finish the assignments.

Designing lesson plans led to a deeper understanding of diseases. The tree-like flowchart allows us to become familiar with emergencies and their management from another perspective. (P22 male)

Strengthened learning by posing questions and challenging each other among peers.(P29 female)

I was very fortunate to participate in the simulation course hosted by Dr. Chiu. (P13)

## Discussion

This study implemented a well-designed, group-developed, and peer-assessed learning approach combined with high-fidelity simulations, an increasingly recognized strategy in medical education. Prior research has shown that PAL enhances engagement and facilitates deeper learning, particularly in simulation-based training where learners actively construct and apply knowledge [7,8]. Our study extends this concept by incorporating a dual-phase PAL model, combining peer-led group discussions with peer-facilitated simulations, an approach that has been less explored in postgraduate emergency medicine training. Similar to findings in studies by Weller et al. (2004) and Steinemann et al. (2011) [1,11], our results suggest that simulation-based PAL fosters teamwork, leadership, and decision-making skills Additionally, our use of reverse selection, where trainees designed cases outside their primary interest, builds upon existing PAL framework by encouraging interdisciplinary learning and adaptive problem-solving [19].

While prior studies highlight that simulation-based training improves resuscitation skills, many trainees still report difficulties in situation monitoring and maintaining composure under pressure, particularly when managing dynamic, high-acuity cases [20,21]. Despite passing ACLS training, participants in our study similarly recognized gaps in their clinical experience, consistent with previous findings that formal certification does not always translate to real-world confidence in critical care settings [22]. However, the high team morale and strong communication and leadership performance observed in our study align with research suggesting that peer-led teams with similar hierarchical levels experience less intimidation and greater collaborative problem-solving. Notably, the three-month post-course evaluation demonstrated sustained improvements in confidence, clinical reasoning, and familiarity with ACLS protocols, reinforcing existing literature on the long-term benefits of simulation-based PAL in knowledge retention and skill application [23].

In this study, the educational program began by forming two groups among the PGY trainees. Within their respective groups, trainees collaborated to collect and discuss clinical cases, enabling them to share knowledge and collectively develop a deeper understanding [24]. When trainees engage in group discussions and debates about various aspects of a clinical case, they are likely to experience deeper cognitive processing. This process involves explaining their reasoning, questioning each other’s understanding, and defending their perspectives, which enhances comprehension and retention of the material [25]. Group discussions also allow trainees to reflect on their own understanding and the reasoning of others, thereby enhancing their metacognitive skills. They learn to evaluate the validity and reliability of different approaches and solutions within a clinical context [26]. During the scenario design phase, peer interactions facilitate testing of the coherence of understanding and solutions. Trainees can challenge each other’s solutions and refine their approaches, leading to a more robust and accurate understanding of clinical scenarios. Ultimately, the accuracy of the students’ final solutions emerges from their interactions and discussions with their peers, rather than solely from their individual knowledge prior to the discussion.

However, while engaging in discussions and debates enhances cognitive processing, it may also increase cognitive load, particularly for trainees with less experience [20]. Cognitive Load Theory suggests that excess cognitive demand can overwhelm working memory and hinder learning efficiency [27]. Engaging in discussions and debates can deepen cognitive thinking and improve problem-solving skills, but it also places additional cognitive demands on trainees, potentially leading to cognitive overload. To mitigate this, our study incorporates structured mentor guidance and scenario-based learning to help trainees manage cognitive load effectively. By balancing challenging discussions with structured debriefing, we aimed to optimize cognitive engagement while preventing overload. Future research could further explore optimal ways to balance cognitive engagement with workload management in peer-assisted simulation training.

The benefits of grouping trainees before PAL in simulation are multifaceted. First, enhanced engagement and motivation: Grouping trainees fosters a sense of community and collective responsibility, which can boost motivation as each member actively contributes to the group’s learning experience [28]. Second, error correction and learning from mistakes: By discussing and testing different scenarios within a group, trainees are more likely to identify and rectify mistakes. The group setting provides immediate feedback and facilitates collective problem-solving, reducing the chances of persisting in incorrect assumptions or approaches [29]. Third, preparation for real-world collaboration: Effective teamwork is essential in clinical settings. Early exposure to collaborative learning equips trainees for their future professional roles, for which multidisciplinary teamwork is often critical to successful patient care [21]. As a result, our trainees exhibited superior performance in leadership, team communication, and team morale during the simulation compared to other competencies.

The flipped classroom is a pedagogical model emphasizing pre-class activities while replacing traditional lectures with student-centered activities [30]. Preparatory activities may include readings, videos, podcasts, or games. During face-to-face sessions, pre-acquired knowledge is applied through case-based discussions, peer instruction, collaborative learning, or simulations. This program uses scenario establishment as the experimental site for the flipped classroom. If simulation objectives suit the flipped model, all activities, including preparatory work and simulations, must align with overall learning objectives. Such alignment highlights key objectives, establishes relevant baseline knowledge, and dedicates face-to-face time to higher cognitive engagement. Dong et al. suggested five recommendations for integrating the flipped classroom model into simulation sessions [31]. They recommended that [1] the learning objectives should dictate the use of simulation in a flipped model [2]; preparation work should be engaging and optimized by applying techniques emphasized in the flipped classroom approach [3]; students should be held accountable for completion of preparation work [4]; flipped simulation should not stand alone but instead be integrated into the larger curricula; and [5] applying the flipped model to simulation requires buy-in from faculty, administration, and students The design of the education plan in our PAL simulation courses fulfilled the recommendations suggested by Dong et al. to strengthen and embrace the learning outcomes of the flipped classroom in simulation.

## Flipped simulation

The value of flipped simulation and student-led simulation is evident in the trainees’ performance. The PAL approach enabled PGY trainees to enhance their learning by posing questions and challenging each other. Additionally, they developed teamwork skills through collaboration with group members. Compared to traditional bedside teaching, simulation-based medical education provides a controlled and safe environment for learning by allowing students to practice managing rare or critical clinical scenarios without risk and to apply and verify textbook knowledge in a practical setting [32,33]. This method also enhances medical expertise and teamwork skills [34]. However, simulation-based education may not adequately stimulate students’ motivation to learn [35]. Our teaching experience corroborates this observation, particularly among students who do not intend to pursue a career in critical care: it is difficult to stimulate their motivation for learning. Physicians are motivated to continue learning and developing their skills because they find the work fulfilling and meaningful, which enhances their sense of self-worth and job satisfaction [22].

Integrating PAL with simulation education has shown numerous benefits, with peer teachers often experiencing greater learning gains than peer learners [9]. Our survey of PGY trainees supports this finding as they provided positive feedback on the process of preparing scenarios. This preparation allowed them to engage in deep reflection on patient cases and to gain a more comprehensive understanding of diseases. Murakami et al. involved medical students acting as facilitators in simulation-based training and debriefing sessions, which enhanced their teaching, teamwork, leadership, and communication skills [36]. Similarly, Kayser et al. combined simulation-based education with PAL for first-year medical residents that focused on providing the students with the necessary competencies to deliver lectures and facilitate workshops [19]. Their study concluded that student facilitators involved in the co-development, delivery, and implementation of scenarios developed both professionally and personally.

## Integrating the flipped classroom model into PAL Simulation-Based Education

The implementation of the flipped classroom model in this PAL simulation curriculum significantly enhanced the trainees’ engagement, preparation, and learning outcomes. By integrating preparatory activities with simulation-based education, the flipped classroom model aligns pre-simulation work with specific learning objectives, thereby ensuring that in-class activities focus on higher levels of cognitive engagement. This design not only optimized the trainees’ understanding of the material but also allowed them to apply theoretical knowledge in practical, real-world scenarios. The step-by-step application of the flipped classroom model in this program is summarized in Table 3, which outlines the methods used to prepare, implement, and evaluate the curriculum. Key strategies included requiring trainees to document clinical observations, develop lesson plans collaboratively, and participate in high-fidelity simulations as facilitators. These steps ensured that the trainees actively engaged in the learning process and took ownership of their education. Moreover, integrating the flipped classroom model within the broader curriculum fostered continuity in learning and strengthened the trainees’ ability to reflect on and apply their knowledge in clinical settings.

**Table 3.** The application of flipped classroom model step-by-step in peer-assisted learning (PAL) simulation.

Steps and recommendation	Methods
Step 1: The learning objectives should dictate the use of simulation in a flipped model	All participants received an course introduction on the first day. The 3-trainee groups were built.
Step 2: Preparation work should be engaging and optimized by applying techniques emphasized in the flipped classroom approach	The simulation scenarios were prepared and recorded from clinical environments where they encountered. Each group discussed all the cases they documented with instructors, and selected 3–4 cases they deemed educationally significant for physicians. (Clinical observation)
Step 3: Students should be held accountable for completion of preparation work	Under instructors’ supervision and consultation, each group selected the most suitable case for developing a teaching scenario. They collaboratively developed lesson plan and comprehension of key learning objectives. (lesion plan development)
Step 4: Flipped simulation should not stand alone but instead integrate into the larger curricula	On the last day of each one-month PGY course, a full afternoon of high-fidelity emergency simulation drills was conducted. The trainees acted as facilitators for the simulation, challenging each other with questions and conducting the scenarios. The trainees also needed to give the feedback to their peers, and they also needed to prepare a 5 mins presentation to express their ideas and hardcore knowledge.
Step 5: Applying the flipped model to simulation requires buy-in from faculty, administration, and students	The trainees were required to complete reflective essay and satisfaction survey on one week later and a clinical proficiency evaluation scale at tree week later for self-reported behavior changes in their future clinical practice. An interview was also conducted for a more substantial understanding of the influence after PAL simulation.

This structured approach underscores the value of combining PAL with flipped classroom strategies to enhance simulation-based education. The alignment of learning objectives, preparatory work, and active simulation exercises not only improved trainees’ clinical competence but also cultivated essential skills such as teamwork, communication, and critical thinking. Future iterations of this program could further refine these methods and explore their applicability in other areas of postgraduate medical training.

## Limitations

This study has several limitations. The small sample size (29 participants) and single-institution setting limit generalizability. Selection bias may have occurred, as motivated trainees with higher baseline competence were more likely to participate. Reliance on self-reported data, such as the PGY-CPE scale, raises the possibility of social desirability bias, while qualitative data from reflective essays may not capture all perspectives. The long-term impact on clinical practice and patient outcomes was not assessed. Future studies with larger, more diverse samples and extended follow-up are needed to validate and expand upon these findings.

## Conclusion

The present findings suggest that the reported curriculum effectively enhanced key competencies essential for clinical practice among PGY physicians. The integration of PAL with high-fidelity simulation training significantly improved trainees’ confidence, reasoning ability, teamwork dynamics, and familiarity with resuscitation procedures. Quantitative data from the TEAM and Ottawa GRS scales showed notable improvements in various domains of clinical performance, whereas qualitative feedback from reflective essays underscored the perceived value of hands-on simulation training and peer learning. This study highlights the importance of incorporating PAL into medical education to bridge the gap between theoretical knowledge and practical application, ultimately fostering a more competent and confident healthcare workforce.

## Supplementary Material

Supplemental Material
