# Supplementary material for: Peer-assisted learning in critical care: a simulation-based approach for postgraduate medical training
Source: Med Educ Online. 2025 May 5;30(1):2497333. doi: 10.1080/10872981.2025.2497333 (PMC12057776; doi:10.1080/10872981.2025.2497333)
Supplement: Supplemental Material [file ZMEO_A_2497333_SM1008.docx]

**Guidance for PGY Trainees in Peer-Assisted Simulation Training**

**Step 1: Group Assignment**

Each month, approximately 6–8 PGY trainees rotate in the ED. You are evenly divided into two groups, each consisting of 3–4 trainees. Group assignment is based on your future specialty preferences: those with a greater interest in surgical specialties are placed in one group, while those inclined toward internal medicine are placed in the other. Within each group, one trainee is designated as the team leader to ensure the smooth operation of the subsequent training sessions.

**Step 2: Case Collection**

During the first two weeks of the ED rotation, each trainee is required to actively identify and collect 1–2 clinical cases that you find educationally meaningful. These cases are prepared in the format of a case-based study. Through group discussions, each team selects three cases, which are then reviewed with an ED faculty mentor to determine their suitability for PGY-level learning or potential adaptation into simulation scenarios.

**Step 3: Case Selection**

With guidance from the faculty mentor, each group ultimately selects one case for structured case development and subsequent simulation training. Trainees in the internal medicine-focused group develop a trauma-related case, while those in the surgical group develop an internal medicine-related case. This approach ensures cross-disciplinary learning, allowing trainees to gain insights into areas outside their primary specialty of interest.

**Step 4: Case Development**

Trainees begin by structuring their case based on **Advanced Cardiovascular Life Support (ACLS)** or **Advanced Trauma Life Support (ATLS)** principles. They simplify the real case into a structured, easily recognizable sequence of clinical events.
**For Examples:**

- **Chest pain** → **Pulse ventricular tachycardia** → **Pulseless ventricular tachycardia** → **Asystole** → **Return of Spontaneous Circulation (ROSC)**
- **High-altitude fall** → **Facial bone trauma with airway obstruction** → **Hemoperitoneum with profound shock** → **Emergency surgery**

Trainees then follow a standardized template to organize the case based on sequential changes in the patient’s clinical status. Each event in the case progression must include:

- **Patient condition**
- **Vital signs**
- **Physical examination findings**
- **Laboratory and imaging results**
- **Interventions performed**
- **Expected clinical response**
- **Learning Point**

This structured approach ensures comprehensive and systematic case-based learning while facilitating the development of high-quality simulation training materials.

**Step 5: Practical Simulation Training**

On the last day of each month, the **PGY Learning Day** for trainees who rotate in ER takes place.

During this afternoon session, trainees participate in TRM simulation. On this day, the internal medicine group PGY trainees present their surgery-based case scenario to assess the surgical group trainees, and vice versa. This cross-disciplinary approach allows trainees to test each other using the cases they have developed.

Trainees take on different roles during the simulation, including:

- **Patient role** (acted out by a trainee)
- **Control room operator (Facilitator)**, responsible for adjusting the **SimMan manikin** and modifying vital signs

Once both groups have completed their simulations, a **one-hour debriefing and discussion session** follows. This session includes:

- **Review of key learning points**
- **Questionnaire and feedback collection**
- **Reflection and experience sharing**

This structured approach ensures comprehensive learning experience and enhances clinical decision-making skills in emergency settings.

Template for Peer Assisted Simulation Training Course

The following Case was the template case for PGY trainees to understand how to transform a true case into a simulation script

Scene: ED Moderate Acuity Area

| Information for Trainees |
| --- |
| Triage Information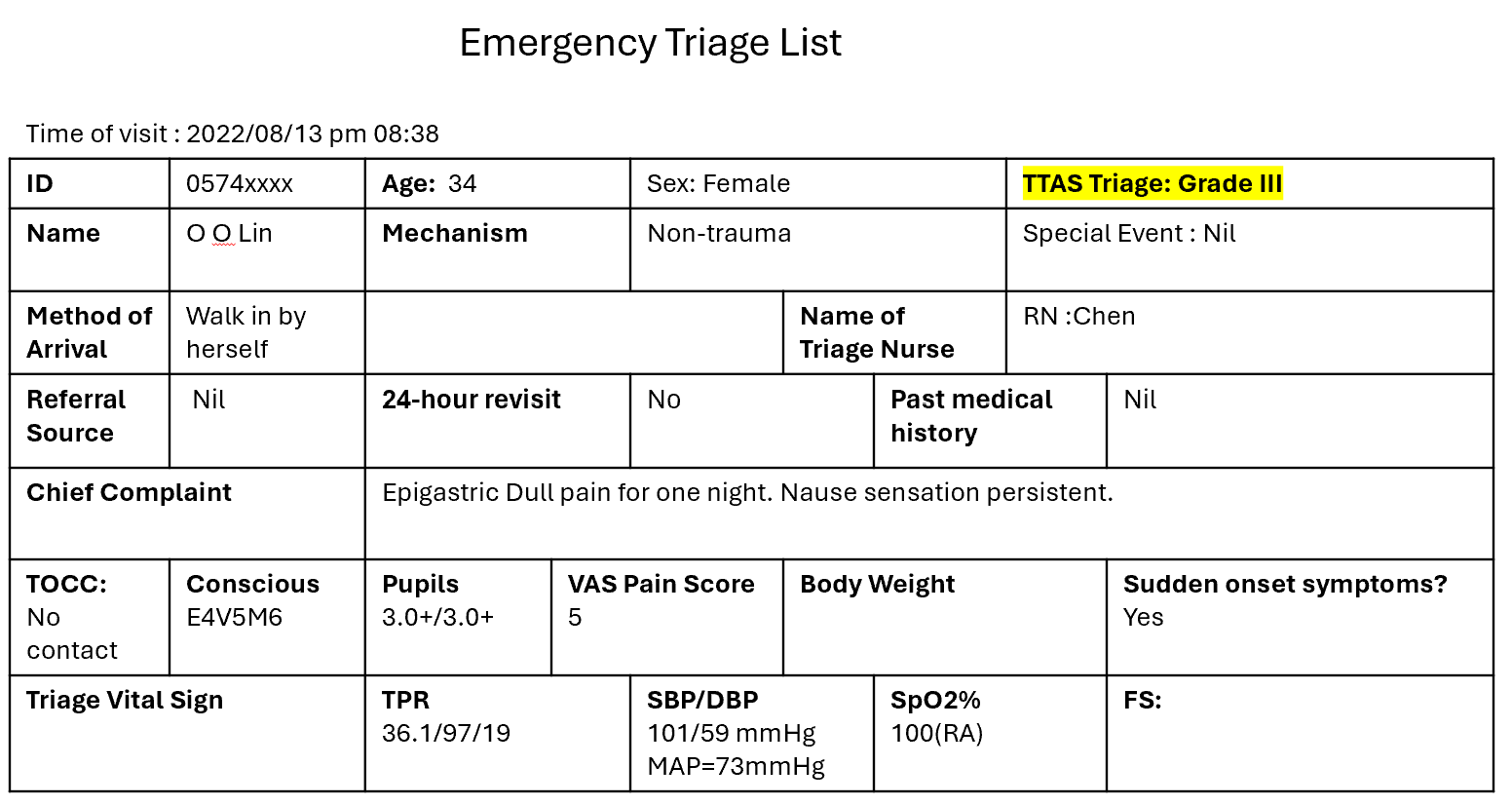 |

# Information for Scenario Announcement:

C.C：acute onset of epigastric dull sensation

Present illness：

resting onset of epigastric dull pain for 1 week, lasting for about 5 minutes in each episode and the frequency increased by time

**Recording**

Team Leader Name: Dr.__________

Number of the team members:

Assessment by : Dr. ____________

**Summarizing the entire clinical sequence, such as:**

Chest pain🡪 ACS 🡪 Pulse VT 🡪 Pulseless VT 🡪 Asystole 🡪 ROSC

**Roles for the script**

Patient (actor name); Consultant (actor name); facilitator (Faculty name)

**Roles within the team**

Leader, Compressor, IV and Medication Administrator, Airway Manager

**#The event begins when the team starts approaching the patient.**

| **Event** | **Patient**  **Condition** | **Vital Sign** | **Physical Exam**  **Findings** | **Lab and Image Results** | **Expected clinical performance** | **Intervention and Management** | **Learning**  **Point** |
| --- | --- | --- | --- | --- | --- | --- | --- |
| I | Oriented.  Can answer questions. |  |  |  | **Leader** |  |  |
|  | Patient had:  Epigastric pain with nausea  Mild tightness  No dyspnea | T: 36.1  P: 97  R: 19  BP: 101/59 | Soft and no obvious tenderness.  Breath Sound clear | No Lab  would be given at current stage | 1.Hands off with triage nurse  2.Taking history  3.Role assignment  4.Arrange ECG Lab and image survey | Pain control  PPI  Primperan  NTG (if they consider ACS) | **Criteria of Triage ECG** |
|  |  |  |  |  | **Team** |  |  |
|  |  |  |  |  | 1.Taking history  2.Initiate Peripheral IV Drip  3.Give medications for symptoms |  |  |

| **Event** | **Patient**  **Condition** | **Vital Sign** | **Physical Exam**  **Findings** | **Lab and Image Results** | **Expected clinical performance** | **Intervention and Management** | **Learning**  **Point** |
| --- | --- | --- | --- | --- | --- | --- | --- |
| II | During  Performing  ECG |  |  | **As attachment File1** | **Leader** |  |  |
|  | Patient started feeling dizziness and dyspnea. | Cons clear  E4V5M6  T: 36.5  P: 94  R: 22  BP: 85/54  SpO2: 97(RA) | Chest：shallow rapid breath  Limbs:  Cold. | Give them lab data if they asked (The elevated cardiac enzyme)  Give them ECG  Give them image if they ask | Role Assignment  Huddle to initiate the new situation  Consider consult CV man  **According to Lab and ECG, ACS or should be the differential diagnosis.**  Notice the possible of ACS and Cardiogenic shock | NSTEMI:   1. DAPT:   Aspirin 100mg/tab 3 tab 75mg/tab (Plavix) 4 tab   1. **Heparinization:**   Loading:  Heparin 25000U/5mL/vial  3600 U; then continue with  Heparin   1. Perform POCUS and management cardiogenic shock | **The treatment of ACS** |

| **Event** | **Patient**  **Condition** | **Vital Sign** | **Physical Exam**  **Findings** | **Lab and Image Results** | **Expected clinical performance** | **Intervention and Management** | **Learning Point** |
| --- | --- | --- | --- | --- | --- | --- | --- |
| III | Conscious change with moaning and | E3V3M5  BP: 70/50  HR:200 ECG-> pulse VT | Pulse (+)  But Profound Shock status | **Monitor showed VT**  **If they ask, give them**  **ABG** | **Leader** | 1. Sync. DC Shock 100J | **Unstable Tachycardia** |
|  |  |  |  |  | 1. Notice the rhythm changed, and recheck pulse 2. ACLS protocol 3. Initiate the resuscitation including cardioversion (know the correct energy for cardioversion) |  |  |
|  |  |  |  |  | **Team** |  |  |
|  |  |  |  |  | 1. Situation monitor 2. Restart Resuscitation 3. Organized the teamwork |  |  |
| IV | Unconscious  Set SpO2= 0 | COMA ECG Monitor 🡪pulseless VT |  | **Ultrasound** (VT, no pericardial effusion, no D shape, bilateral lung sliding(+), IVC>2cm)  **Capnography (if they apply)** (ETCO2: 3->re-on 23) | **Leader & Team** | 1. Defib. DC shock 200J 2. Epinephrine 3. Amiodarone 4. ETT 5. Consider ECMO | **Cardiac Arrest Protocol** |
|  |  |  |  |  | 1. Initiate CPCR 2. ACLS protocol 3. Role assignment 4. Check 5H5T 5. Consider ECPR(ECMO) |  |  |
|  |  |  |  |  | **TRM**   1. How the situation monitor present in the case. 2. How the team huddle when new scenario happened |  |  |
| V | Cardiac arrest | ECG monitor  🡪Asystole |  | **Capnography** | **Team** | 1. Epinephrine and keep CPCR. 2. Monitor the quality of CPR (CPR coach) | **-5H5T**  **-CPR coach**  **-Timing for ECPR** |
|  |  |  |  |  | 1. Run a high-performance team 2. Consultant CVS surgeon for the ECPR |  |  |

| Attach  File  Lab | **Complete Blood Count (CBC)**  **And Coagulation Profile**   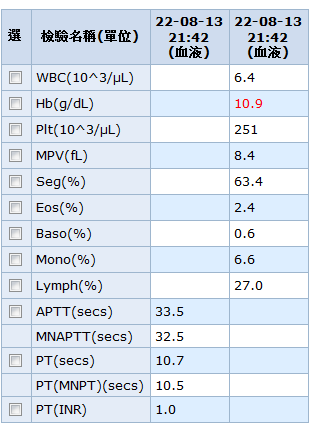 *Red-colored values indicate abnormal results.* | **Biochemistry Panel** 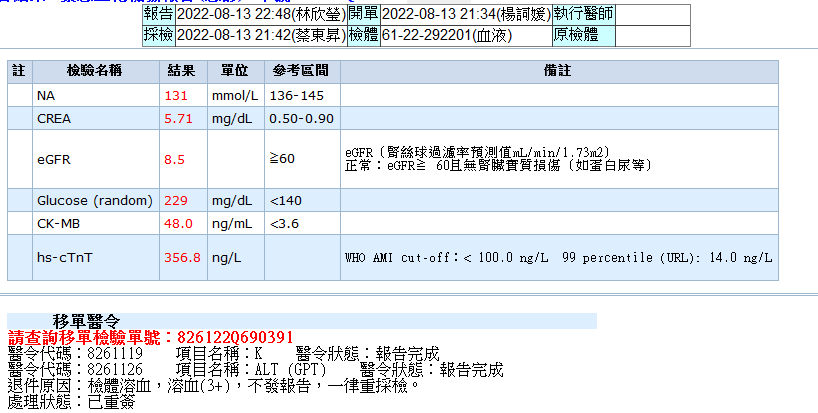 *Red-colored values indicate abnormal results.* | V**ein Gas Analysis** 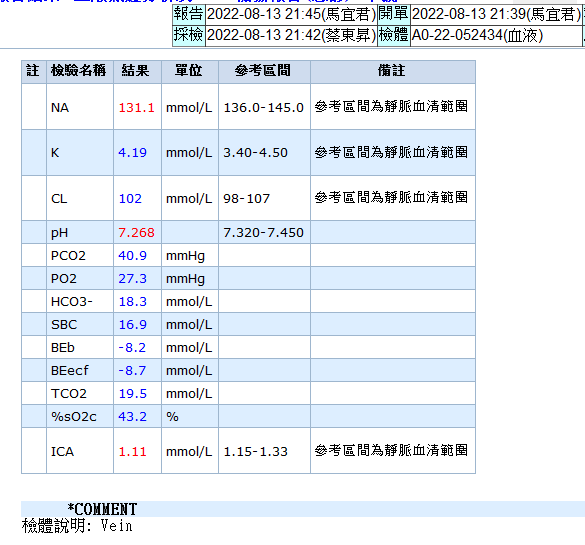 *Red-colored values indicate abnormal results.* |
| --- | --- | --- | --- |
| Attach  File  ECG | 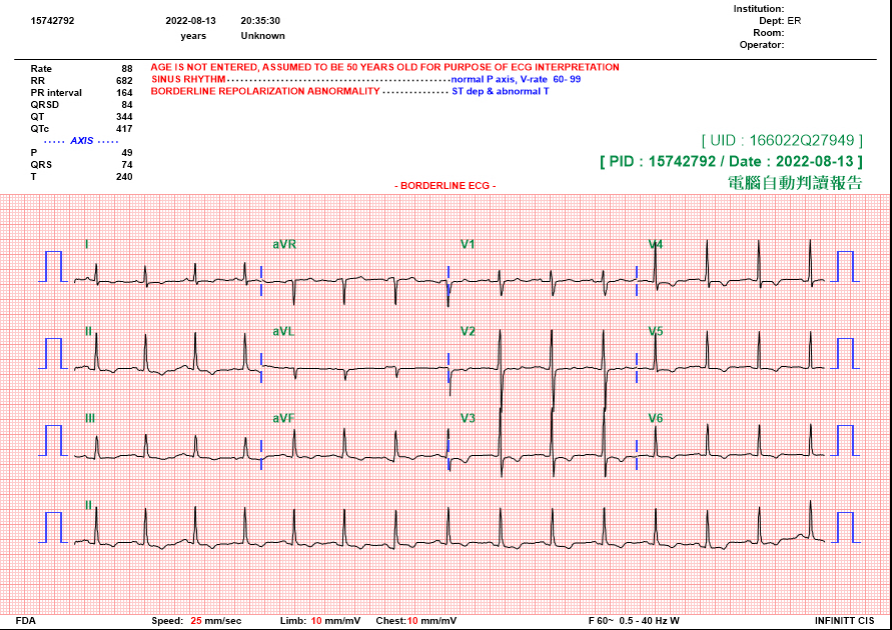 | | |
| Attach File  XRAY | 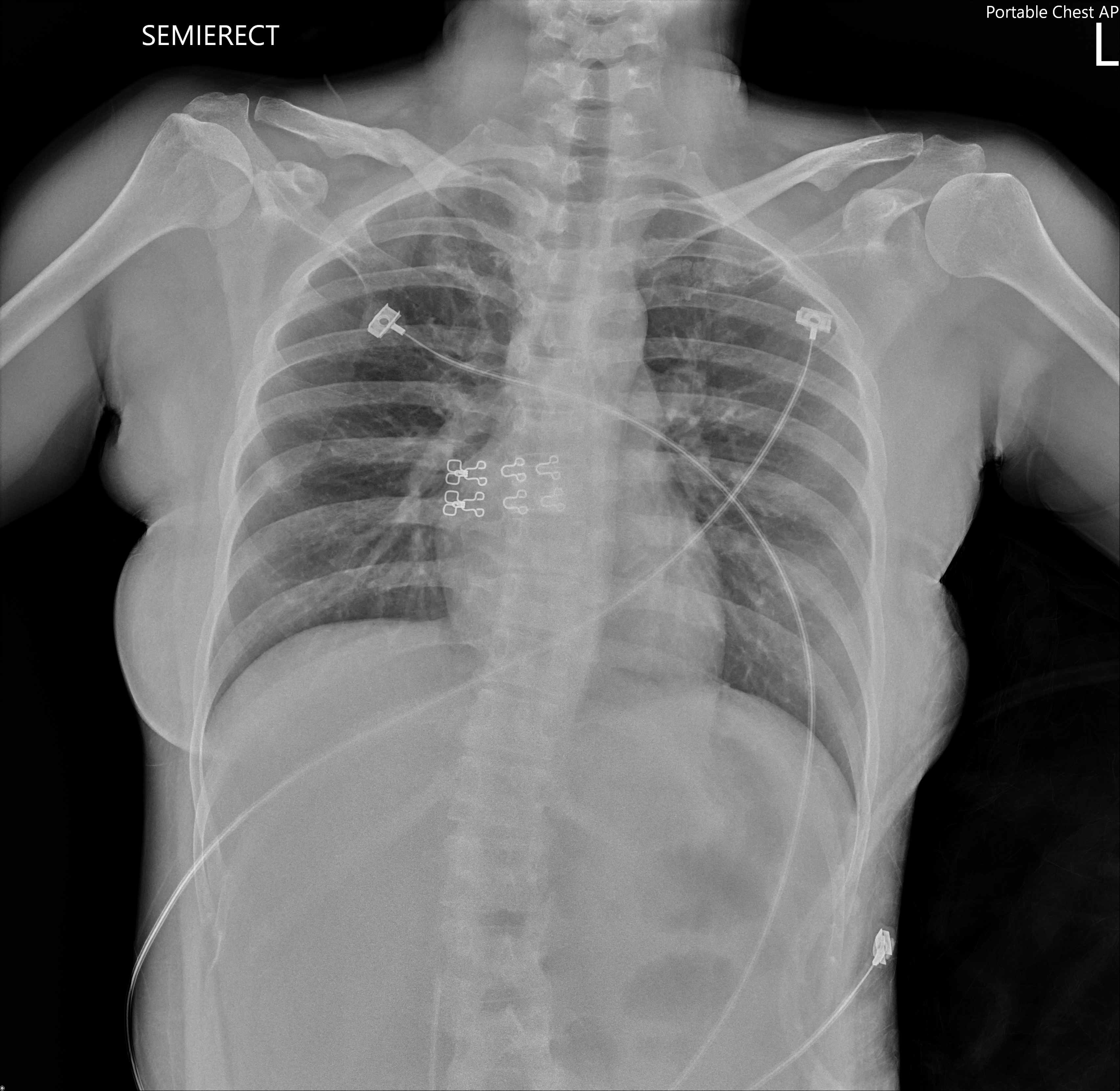 | | |

**Appendix: Abbreviations and Color Coding**

**Abbreviations**

**ACS** – Acute Coronary Syndrome

**APTT** – Activated Partial Thromboplastin Time

**ATLS** – Advanced Trauma Life Support

**BP** – Blood Pressure

**CVS** – Cardiovascular Surgeon

**DAPT** – Dual Antiplatelet Therapy

**ECG** – Electrocardiogram

**ECMO** – Extracorporeal Membrane Oxygenation

**ETCO₂** – End-Tidal Carbon Dioxide

**ECPR** – Extracorporeal Cardiopulmonary Resuscitation

**IV** – Intravenous

**MNAPTT** – Mean Normal Activated Partial Thromboplastin Time

**NTG** – Nitroglycerin

**PPI** – Proton Pump Inhibitor

**PT** – Prothrombin Time

**RA** – Room Air

**ROSC** – Return of Spontaneous Circulation

**SpO₂** – Peripheral Capillary Oxygen Saturation

**TRM** – Team Resource Management

**TTAS** – Taiwan Triage and Acuity Scale

**VT** – Ventricular Tachycardia

**Color Coding Key**

**Red-colored values** (e.g., “10.9”) indicate abnormal findings that require immediate medical intervention.

**Black-colored values** are within normal limits and serve as clinical baselines or stable parameters.

**Yellow-highlighted text** represents moderate-acuity (Level III) patients according to our institution’s triage system.
In our emergency department, patients are triaged into three levels based on urgency:

Red: High-acuity (Level I–II), requiring immediate attention

Yellow: Moderate-acuity (Level III), requiring timely but not immediate intervention

Green: Low-acuity (Level IV–V), stable patients with non-urgent conditions

**Chinese Translation**

**檢驗名稱(單位): Test Name (Unit)**

**血球檢驗: Complete Blood Count (CBC)**

**生化檢驗: Biochemical Tests 或 Biochemistry Panel**

**參考區間: Reference Range**

**結果: Result**
